# AGEs Secreted by Bacteria Are Involved in the Inflammatory Response

**DOI:** 10.1371/journal.pone.0017974

**Published:** 2011-03-22

**Authors:** Ifat Cohen-Or, Chen Katz, Eliora Z. Ron

**Affiliations:** Department of Molecular Microbiology and Biotechnology, Faculty of Life Sciences, Tel Aviv University, Tel Aviv, Israel; Agency for Science, Technology and Research (A*STAR), Singapore

## Abstract

Advanced Glycated End Products (AGEs) are formed by non-enzymatic protein glycation and are implicated in several physiological aspects including cell aging and diseases. Recent data indicate that bacteria – although short lived – produce, metabolize and accumulate AGEs. Here we show that *Escherichia coli* cells secret AGEs by the energy-dependent efflux pump systems. Moreover, we show that in the presence of these AGEs there is an upshift of pro-inflammatory cytokins by mammalian cells. Thus, we propose that secretion of AGEs by bacteria is a novel avenue of bacterial-induced inflammation which is potentially important in the pathophysiology of bacterial infections. Moreover, the sensing of AGEs by the host cells may constitute a warning system for the presence of bacteria.

## Introduction

Protein glycation is a process in which reducing sugars interact with primary amines on the side chains of lysine and arginine, resulting in a chemical sequence of reactions known as “Amadori rearrangement” which leads to the formation of Amadori-modified proteins (AMPs). AMPs are reversible intermediates of glycation processes, and several mechanisms involved in their catabolism have been described [1], [2], [3], [4], [5]. However, AMPs can further developed, in an oxidation-dependent manner, to form irreversible, highly stable compounds known as Advanced Glycation End-products (AGEs).

AGEs were shown to participate in the pathophysiology of several age-related diseases [6], [7], [8], [9]. They interact with specific receptors which mediate intracellular signaling that leads to enhanced oxidative stress and elaboration of key pro-inflammatory cytokines [3], [10], [11], [12]. Several AGEs receptors have been identified, including macrophage scavenger receptors Types I and II, and are expressed on a wide range of cells [13]. Of special interest is RAGE (Receptor for AGEs) which is involved in inflammation and sepsis. Thus, reduction of RAGE activity by genetic manipulations or anti-RAGE antibodies, was shown to reduce inflammation and protects from sepsis in a murine model [14], [15].

AGEs also exist in bacteria [16] and in *E. coli* they are metabolized and accumulate as low molecular weight compounds [17]. Here we show that *E. coli* cells secrete AGEs by an active, energy-dependent system which is carried out by the efflux pumps system. Moreover, we show that the secreted AGEs participate in the inflammation response of mammalian cell cultures, indicating that they play a role in the phatophisiology of bacterial infections.

## Results

### AGEs are secreted

**Figure 1:**
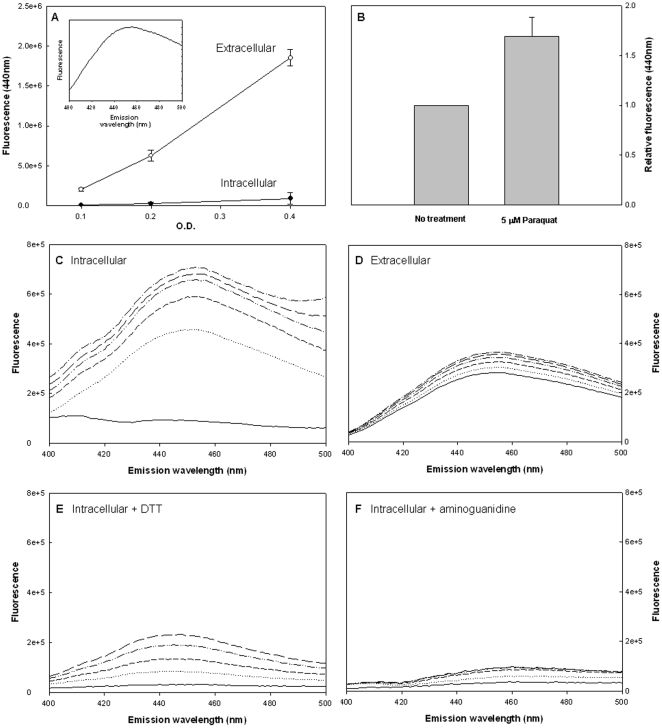
Kinetic studies of AGEs formation and secretion. Bacterial growth and sample collection were described in materials and methods. AGEs-specific fluorescence (Ex. 370, Em. 440) was determined and normalized to cells density. A) AGEs-specific fluorescence during growth in the intracellular and extracellular fractions. The insert shows the distribution of AGEs with excitation at 370 nm. B) Effect of 5 µM paraquat on Extracellular accumulation of AGEs. C–D) Kinetics of AGEs formation in vitro. Bacteria were separated from extracellular fractions and sonicated (intracellular fraction). C) The intracellular and D) extracellular fractions were incubated at 37°C and AGEs-specific fluorescence was determined every 30 minutes (Ex. 370, Em. 440–500). Effect of E) 2 mM DTT or F) 50 mM aminoguanidine on AGES in the intracellular fractions. All data represent three independent experiments, in Figures C–F a representative result is shown.

The intracellular concentrations of AGEs are relatively steady, but with time there is a dramatic accumulation of AGEs in the medium, (Figure 1A). This finding is based on monitoring the fluorescent fraction of secreted AGEs, which has the typical peak at 440 nm and a broad spectrum, reflecting the heterogeneity of the fraction (see insert of Figure 1A). Based on size-exclusion filtration we determined that the extracellular AGEs are smaller than 3 kD. This finding is compatible with the assumption that they originate from catabolism of protein glycation products [17]. The concentration of AGEs in the supernatant are considerably higher than these of the intracellular AGEs, suggesting that the intracellular concentration is maintained constant by secreting the excess. This assumption is further supported by the finding that the secretion of AGEs is enhanced under oxidative conditions, which stimulates the oxidation-dependent formation of AGEs. Thus, the addition of the oxidative agent *N,N′-*dimethyl-4,4′-bipyridinium dichloride (Paraquat, Sigma) resulted in a significant increase in secreted AGEs (Figure 1B).

### AGEs are secreted in their mature form

The presence of AGEs in the medium can result from a secretion process of AGEs which are formed inside the bacteria, alternatively, it is possible that the intermediates of AGEs are secreted and the formation of AGEs is taking place outside the cells. To distinguish between these two possibilities we determined the potential of AGEs formation in the intracellular and extracellular fractions, taking advantage of the fact that fluorescent AGEs are formed by cross-linking of corresponding Amadori-products intermediates. Bacterial lysates and secreted extracellular fractions were incubated at 37°C for 2.5 hours in PBS buffer and AGEs-specific fluorescence was measured every 30 minutes.

In the intracellular fraction (bacterial lysates), the AGEs-specific fluorescence increased with time reaching over 6 fold of the basal level. In contrast, in the extracellular fraction, fluorescence increased was minimal (Figure 1C+D). As a control, DTT was added to the intracellular fraction to prevent development of AGEs, since AGEs formation is oxidation depended. Indeed, addition of 2 mM DTT resulted in a considerable decrease in the rate of AGEs formation (Figure 1E) Moreover, addition of 50 mM aminoguanidine, an agent known to prevent the essential cross-linking in AGEs formation [18] completely abolished AGEs accumulation (Figure 1F). These results demonstrate that intermediates of AGEs formation are restricted to the intracellular fraction and that the secreted molecules are “mature” AGEs.

### Accumulation of AGEs requires protein synthesis and energy

**Figure 2:**
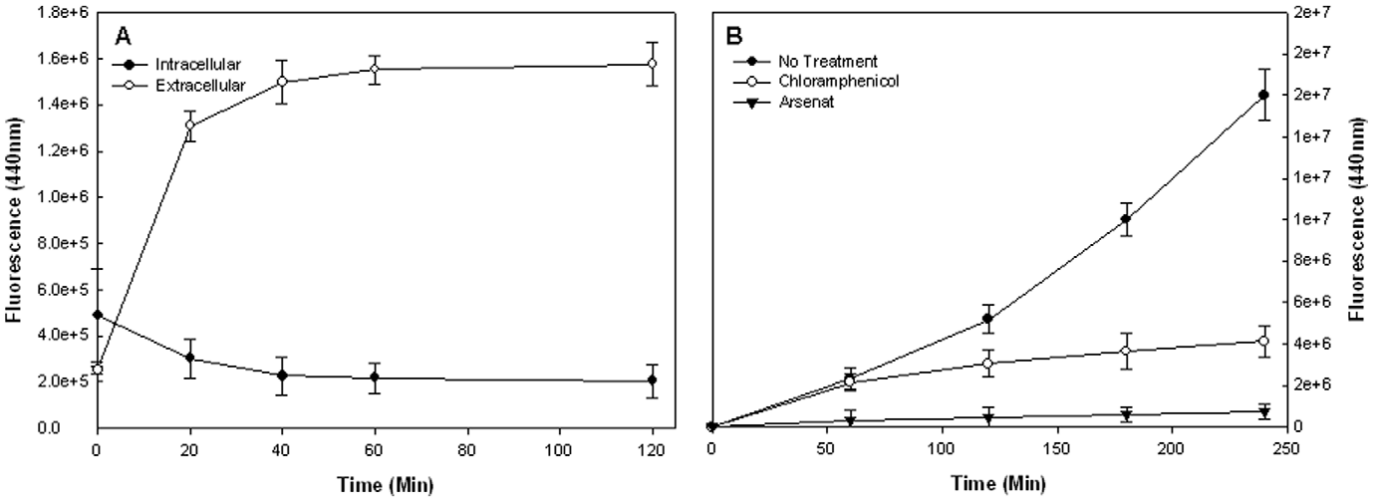
Effect of protein synthesis arrest on AGEs secretion. Bacteria were grown in MOPS minimal medium and samples were collected for AGEs determination as described in materials and methods. AGEs-specific fluorescence (Ex. 370, Em. 440) was determined and normalized to cells density. A) Intracellular and extracellular AGEs levels following exposure to chloramphenicol. B) Effect of chloramphenicol (open circles) and Arsenate (triangles) on AGEs secretion kinetics. The data represent three independent experiments.

AGEs are the end product of protein glycation processes. In order to determine whether the secretion of AGEs is dependent on protein synthesis we examined the effect of a translational arrest on the kinetics of AGEs secretion. The results presented in Figure 2A demonstrate that following translational arrest by chloramphenicol the intracellular concentration of AGEs is depleted. Concurrently, the concentration of AGEs in the extracellular fraction increases, suggesting that production of AGEs, but not their secretion, is blocked in the absence of protein synthesis. In the presence of chloramphenicol the accumulation of extracellular AGEs reaches a plateau, which is at a lower value than without translational arrest, indicating that new AGEs are not formed in the absence of translation (Figure 2B).

An even more drastic decrease in the level of AGEs in the medium was obtained when arsenate was added to deplete internal ATP levels (Figure 2B). Part of this effect is due to the requirement of ATP for protein synthesis. However, the finding that ATP depletion has a stronger effect than a translational arrest indicates the involvement of additional energy dependent processes.

### AGEs are secreted by the efflux pumps system

**Figure 3:**
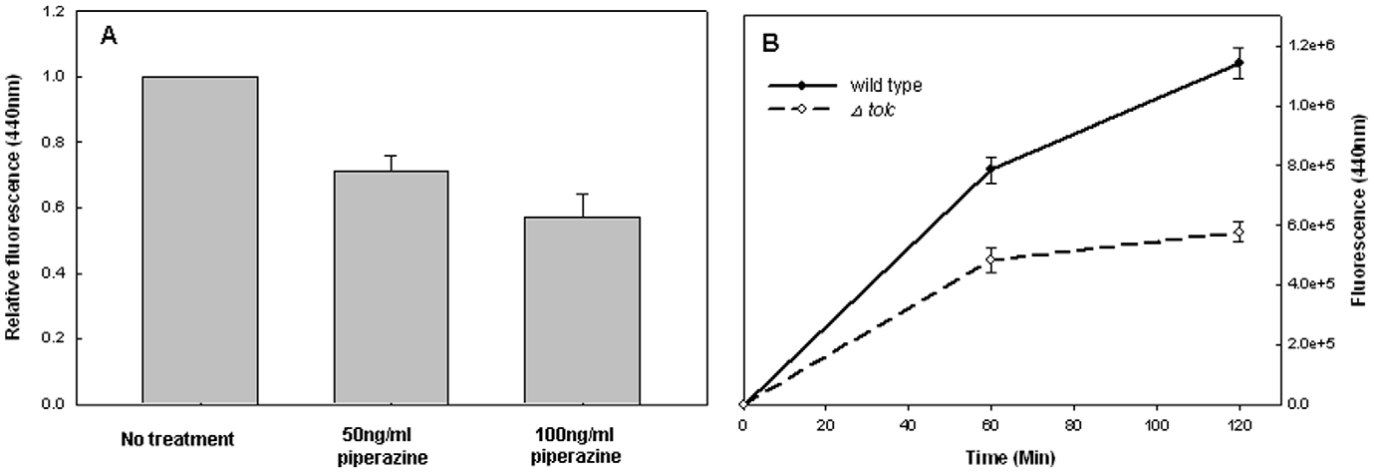
Effect of an efflux pumps inhibitor and *tolC* deletion mutant on AGEs secretion. Effect of A) 50 ng/ml and 100 ng/ml of piperazine or B) *tolC* deletion on the kinetics of AGEs secretion. The data represent three independent experiments.

The finding that AGEs secretion is ATP dependent (Figure 2B), suggests the involvement of an active pumps system in their secretion. Active efflux systems – efflux pumps – are present in all living cells and are responsible for extrusion of toxic substances and antibiotics outside the cell in an energy-dependent manner [3]. The efflux pumps decreases the antibacterial activity of many unrelated drug families and can be considered as a ‘general’ resistance mechanism and thus contribute to bacterial multidrug resistance (MDR) [19], [20]. To examine this possibility we used 1-(1-Naphthylmethyl)-piperazine - an efflux pumps inhibitor that has been shown to reverse multidrug resistance (MDR) in *E. coli* [21], [22]. Indeed, there was a clear concentration-dependent inhibitory effect of the efflux pump inhibitor on secretion of AGEs (Figure 3A).

In order to further examine the rule of the efflux pump systems in the secretion of AGEs, we constructed a *tolC* deletion strain (Δ*tolC*) and studied its effect. TolC is an outer membrane protein known to participate in the activity of wide variety of efflux pumps. TolC is involved in the export of chemically diverse molecules ranging from large protein toxins to small toxic compounds, such as antibiotics [22], [23]. TolC mutants were shown to be highly sensitive to a wide variety of organic compounds demonstrating its secretion deficiency [24], [25]. The Δ*tolC* mutant showed a dramatically reduced secretion of AGEs (Figure 3B) indicating the involvement of TolC in this process.

### AGEs secreted from bacteria cause inflammation in human cells

**Figure 4:**
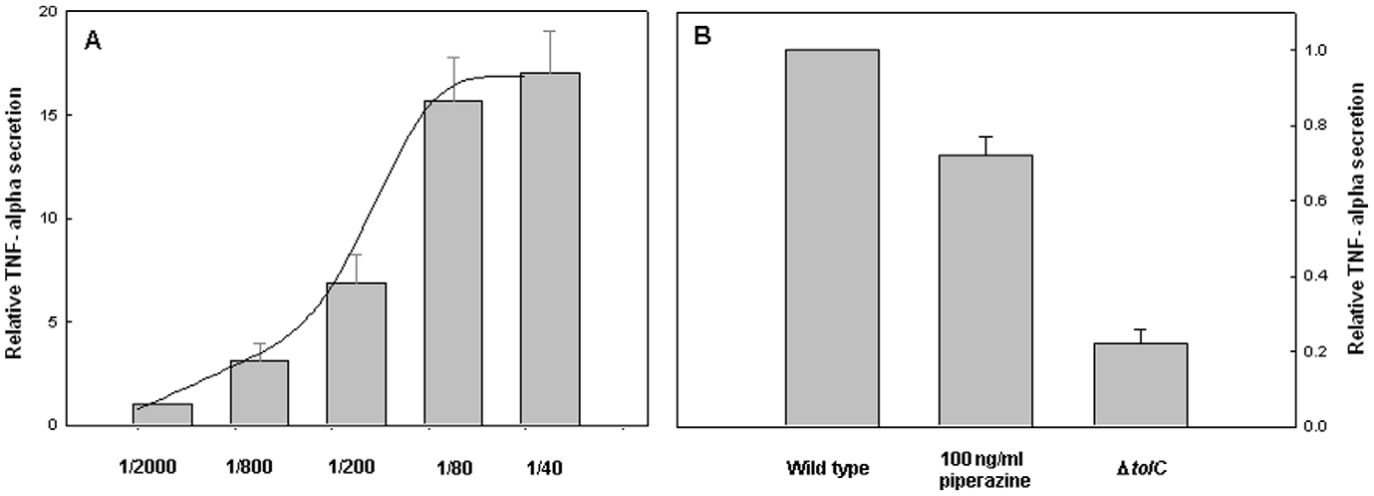
Effect of extracellular AGEs on secretion of TNF-alpha by THP-1 cells. The levels of TNF-alpha secreted by THP-1 cells were measured using sandwich-ELISA (see methods). A) TNF-alpha levels following exposure to elevated concentration of AGEs-containing fraction. B) Relative TNF-alpha levels secreted from THP-1 cells following exposure to the extracellular fractions (1/80 stock dilutions in distilled water) secreted from wild type cells, peprazine-treated cells and Δ*tolc* mutant cells. The data represent three independent experiments.

Endogenous AGEs cause inflammation through interaction with specific receptors (RAGE) [13]. As an example, THP-1 monocytic cells respond to AGEs by production of cytokines [26]. Our findings that bacteria secrete AGEs suggested the possibility that these AGEs can be involved in the inflammation processes. Indeed, exposure of THP-1 cells to AGEs-containing fractions resulted in an increase of TNF-alpha secretion. The magnitude of the inflammatory response was proportional to the levels of AGEs in the fraction (Figure 4A).

The levels of the pro-inflammatory cytokine TNF-alpha were considerably reduced under conditions resulting in reduced secretion of bacterial AGEs, i.e., upon inhibition of the bacterial efflux pumps by peprazine or by deleting the efflux pumps component TolC (Figure 4B). These results demonstrate the strong correlation between the levels of AGEs in the extracellular fraction and THP-1 inflammatory response and emphasize the ability of AGEs secreted by bacteria to induce inflammation in mammalian cells.

## Discussion

The bacterial-host interactions involve a recognition process in which each of the partners senses the presence of the other. This sensing results in a cascade of molecular and biochemical reactions aimed at adapting to the new situation. Here we provide evidence that bacteria secrete protein glycation products (AGEs) which are sensed by mammalian cells and trigger the inflammation cascade.

AGEs are ubiquitous irreversible end products of protein glycation which are formed from Amadouri protein products. We previously demonstrated that AGEs are formed and metabolized in E. coli and that their accumulation is deleterious. Here we show that AGEs are secreted by E. coli, suggesting a mechanism for disposal of these irreversible protein glycation products from the cells. A cellular mechanism responsible for secretion of AGEs in higher organisms has not been described yet, however there are evidence indicating that in diabetic patients, AGEs are excreted in the urine [27], [28], demonstrating a systemic removal of glycation products and probably reflecting a universal need for their removal.

Inhibition of protein synthesis by chloramphenicol dramatically reduced AGEs accumulation in the medium indicating that AGEs formation is dependent on protein synthesis. We hypothesize that under normal growth conditions new proteins are constantly synthesized and glycated, while when the synthesis of new proteins ceased, glycation is limited by existing pool of proteins. When ATP production was disrupted by arsenate, AGEs accumulation in the medium was further reduced, indicating that another ATP dependent mechanism is involves in AGEs formation or secretion. Our finding that the efflux pumps system play a role in this process presents an additional mechanism in which ATP is involved. However it is possible that other processes in the pathway of AGEs synthesis and secretion requires energy.

Inflammation is a cellular response designated to protect the host against invading pathogens. The innate immune system is able to detect pathogens via a limited number of pattern-recognition receptors [29], [30]. In addition, inflammation can also occur in response to intracellular signals. In mammalians, AGEs were shown to mediate intracellular signaling that leads to enhanced oxidative stress and elaboration of key pro-inflammatory cytokines [10], [11], [12], [3].

Exposure of human THP-1 cells to E. *coli* supernatant resulted in induction of cellular inflammation. The level of the inflammatory response was highly correlated with the concentration of AGEs in the medium. This result suggests that AGEs secreted by bacteria may be sensed by mammalian cells, and present a possible model for the involvement of the Receptor for AGEs (RAGE) in bacterial infection and sepsis. Here we suggest a new role for RAGE as a detector of bacterial metabolites and, thus, bacterial presence.

To conclude, we show that AGEs are formed in bacteria as the end product of protein glycation processes and are actively secreted into the medium. We propose that RAGE-expressing cells can detect these AGEs and activate the immune system leading to enhanced inflammatory response. Thus, RAGE may functions as sensor for bacterial invasion.

## Materials and Methods

### Bacterial strains and growth conditions

The *E. coli* K12 wild type strain MG1655 was used throughout. Its *tolC* deletion (Δ*tolC*) was constructed by the one step inactivation system as described previously [31]. Cultures were grown with aeration at 37°C to the exponential growth phase in MOPS minimal medium [32] supplemented with 0.2% glucose.

### Sample preparation

Extracellular fractions were separated from the bacteria using centrifugation and 0.2 µm filtration (Sartorius Stedim biotech) and AGEs were determined in the cells (sonicated) and in the supernatant. When required cultures were treated with 100 µg/ml chloramphenicol (Sigma) or 50 µg/ml arsenate (Sigma). In experiments involving determination of AGEs in non-growing cells (Figure 1B, and Figure 3) cultures were washed and brought to the same turbidity in glucose-free media and incubated for two hours. For determination of inflammation, bacteria (2×10^8^/ml) were washed and incubated for 2 h in glucose free MOPS. AGEs-containing supernatant samples were concentrated 20 fold using Speed-Vac and used as stock solution.

### Determination of AGEs

AGEs were quantified using the natural AGE-specific fluorescence (Ex. 370 nm, Em. 440 nm) by scanning emission ranging from 400 nm to 500 nm upon excitation at 370 nm at 37°C, in a HORIBA scientific FluoroLog-3 Spectrofluorometer. Data represent either the full range spectrum, or the 440 nm emission peak, as indicated in the legends.

### Cell culture medium and growth conditions

The human THP-1 monocytic cells (THP-1) [26] were grown in cell culture medium - RPMI 1640 supplemented with 20% FCS, glutamine (2 mM), streptomycin/penicillin (100 mg/mL/100 U/mL), sodium pyruvate (2 mM) and Non-Essential Amino Acids Solution at 37°C, in a humidified atmosphere of 95% air and 5% CO_2_.

### Inflammation assay

THP-1 cells (2×10^6^ cells/ml) were incubated in 96-well tissue culture plates in serum-free culture medium. Cells were treated with bacteria extracellular stock solution (see sample preparation) in a 1/80 dilution in distilled water or as indicated, for 12 hours. The medium was then harvested and assayed for TNF-alpha levels by sandwich ELISA using 4 µg/ml Anti-Human TNF-alpha (peprotech), 0.5 µg/ml Biotinylated Anti-Human TNF-alpha (peprotech), 0.1 µg/ml Streptavidin- HRP (Enco) and TMB (RandD). Untreated THP-1 cells were used as blank and concentrated MOPS was used as a control.
